# Comparative analysis of changes in retinal layer thickness following femtosecond laser-assisted cataract surgery and conventional cataract surgery

**DOI:** 10.1186/s12886-024-03543-1

**Published:** 2024-07-09

**Authors:** Dongheon Surl, Seungmin Kim, Sangyeop Kim, Tae-Im Kim, Kyoung Yul Seo, Ikhyun Jun

**Affiliations:** 1 The Institute of Vision Research, Department of Ophthalmology, Gangnam Severance Hospital, Yonsei University College of Medicine, Seoul, South Korea; 2 The Institute of Vision Research, Department of Ophthalmology, Yonsei University College of Medicine, Seoul, South Korea; 3 Department of Refractive Surgery, B&VIIT Eye Center, Seoul, South Korea; 4 Clear Eye Clinic, Pyeongtaek-si, Gyeonggi-do South Korea; 5 Corneal Dystrophy Research Institute, Yonsei University College of Medicine, Seoul, South Korea; 6 David H. Koch Institute for Integrative Cancer Research, Massachusetts Institute of Technology, Cambridge, MA USA; 7 Department of Chemical Engineering, Massachusetts Institute of Technology, Cambridge, MA USA

**Keywords:** Femtosecond laser-assisted cataract surgery, Conventional cataract surgery, Retinal layer thickness

## Abstract

**Background:**

To investigate the influence of femtosecond laser-assisted cataract surgery (FLACS) on macula by examining changes in retinal layers after FLACS and to compare these changes with those after conventional cataract surgery (CCS).

**Methods:**

This study included 113 unrelated Korean patients with age-related cataract who underwent CCS or FLACS in Severance Hospital between September 2019 and July 2021. Optical coherence tomography was performed before and 1 month after surgery. The total retinal layer (TRL) was separated into the inner retinal layer (IRL) and outer retinal layer (ORL); moreover, the IRL was subdivided into the retinal nerve fiber layer, ganglion cell layer, inner plexiform layer, inner nuclear layer (INL), outer plexiform layer, and outer nuclear layer. We performed between-group comparisons of the postoperative thickness in each retinal layer and the postoperative differences in retinal thickness. The average retinal thickness of the four inner macular ring quadrants was used for comparative analysis.

**Results:**

Compared with the CCS group, the FLACS group exhibited a thicker ORL (*P* = 0.004) and a thinner INL (*P* = 0.007) after surgery. All retinal layer thickness values showed significant postoperative changes regardless of the type of surgery (*P* < 0.05). The postoperative increase in TRL and IRL thickness was significantly smaller in the FLACS group than in the CCS group (*P* = 0.027, *P* = 0.012).

**Conclusions:**

The 1-month postoperative retinal changes were less pronounced in the FLACS group than in the CCS group.

**Supplementary Information:**

The online version contains supplementary material available at 10.1186/s12886-024-03543-1.

## Introduction

Femtosecond laser-assisted cataract surgery (FLACS) is a widely utilized surgical technique that is replacing conventional cataract surgery (CCS) due to its advantages in the anterior segment [1–7]. Specifically, FLACS has advantages with respect to enhanced precision in the positioning, shape, and size of the capsulotomy, as well as reduced intraocular lens tilt and higher-order aberrations [1–5]. Additionally, using a laser for lens fragmentation leads to a reduced need for ultrasound energy during lens nucleus removal and decreases endothelial cell count (ECC) loss [4, 5]. 

However, there is a relative paucity of reports regarding retinal changes after FLACS. In CCS, there has been extensive research on postoperative cystoid macular edema (CME) and the underlying mechanism. Although the exact cause of CME remains unclear, postoperative inflammatory processes have been proposed as potential triggers [8–14]. After cataract surgery, there is increased synthesis of chemical mediators such as prostaglandin (PG). This causes disruption of the blood-aqueous barrier, leading to increased dispersion of PGs and other cytokines into the aqueous humor and vitreous. Consequently, these inflammatory mediators exacerbate the disruption of the blood-retinal barrier, ultimately increasing the incidence of postoperative CME [8, 12–14].

Schultz et al. found that FLACS increased PG levels in the anterior chamber, [8] which may contribute to macular edema. Furthermore, as the FLACS procedure involves extra maneuvers such as docking or applanation and exposure of the eye to energy sources other than ultrasound, concerns exist regarding the risk of postoperative CME associated with FLACS. Contrastingly, compared with CCS, FLACS may have a lesser surgical impact on macula given its significantly reduced phacoemulsification power and time, [15, 16] which are significant risk factors for macular edema [10, 11].

Reports regarding the impact of FLACS on the macula have been inconsistent; accordingly, further studies are warranted. Elucidation of macular alterations after FLACS and CCS could provide valuable information regarding the effect of femtosecond laser technology on the macular region.

Based on previous research, the most significant macular changes occur approximately 1 month after surgery [17–20]. Accordingly, focusing on the degree of changes during this specific timeframe could allow more distinct between-procedure comparisons of the postoperative effects. Furthermore, examination of each retinal layer after cataract surgery might allow further elucidation of the specific influence of FLACS on the macula. Therefore, we aimed to assess and compare the influence of each surgical approach on the macula by examining changes in retinal layers using optical coherence tomography (OCT) before and one month after FLACS or CCS.

## Methods

### Patient recruitment

This single-center retrospective study included 113 unrelated Korean patients with age-related cataract who underwent CCS or FLACS between September 2019 and July 2021. The patients underwent ophthalmologic examinations, including slit lamp examination; fundus examination; and measurement of corrected distant visual acuity (CDVA), intraocular pressure (IOP), refractive error, and axial length. We investigated the clinical information of each patient, including medical and ophthalmologic history. Phacoemulsification time and cumulative dissipated energy (CDE) values were collected when available.

The exclusion criteria were as follows: age < 20 years and > 80 years; axial length ≤ 21.5 mm or ≥ 26 mm; history of retinal disorder; retinopathy treatment (vitrectomy, laser photocoagulation, intravitreal injection, etc.), or glaucoma; and missing retinal examination due to opacity of the crystalline lens or other disorders. This study was approved by the Institutional Review Board of Severance Hospital, Yonsei University College of Medicine (4-2022-1056), and the need for consent to participate was waived by the board. This study was performed in accordance with the tenets of the Declaration of Helsinki.

### Surgery

All cataract surgeries were performed by a skilled surgeon (I.J). Following pupil dilation and application of topical anesthetics, the subsequent procedures were performed using the femtosecond laser system. The LenSx laser system (Alcon, Aliso Viejo, CA, USA) utilized a curved contact lens to flatten the cornea; further, the position of the crystalline lens surface was determined using OCT. A 4.5-mm diameter capsulotomy procedure was performed by scanning a cylindrical pattern from 100 μm below to 200 μm above the anterior capsule. We applied grid pattern lens fragmentation. The laser created a self-sealing biplanar 2.8-mm corneal incision, followed by 1.0-mm side-port incision.

Upon completion of the femtosecond laser pretreatment, the patient was taken to the main operating room. The self-sealing corneal incisions were carefully opened using a blunt spatula, followed by introduction of viscoelastic material to maintain the anterior chamber. The 4.5-mm diameter capsulotomy site was gently extracted from the eye using rhexis forceps. Hydrodissection was performed, and the lens fragments were removed using the traditional phacoemulsification technique. Next, the lens cortex was removed, and a one-piece, hydrophobic, acrylic posterior chamber lens was introduced. The viscoelastic material was thoroughly removed through irrigation-aspiration.

Patients in the CCS group underwent manual continuous curvilinear capsulorrhexis for capsulotomy and the divide and conquer technique for lens fragmentation. CDE and phacoemulsification time was measured after each surgery, if available.

Except for mild subconjunctival hemorrhage, no intraoperative or postoperative complications were observed in either group. During the first postoperative month, topical antibiotic and steroid eye drops were applied four times a day, while topical nonsteroidal anti-inflammatory drug eye drops were applied two times a day.

### Measurement of retinal layer thickness

OCT (Heidelberg Engineering, Heidelberg, Germany) was performed before and 1 month after surgery. All patients underwent OCT in the early afternoon of the day during their outpatient visit. The total retinal layer (TRL) was separated into the inner retinal layer (IRL) and outer retinal layer (ORL); further, the IRL was subdivided into the retinal nerve fiber layer (RNFL), ganglion cell layer (GCL), inner plexiform layer (IPL), inner nuclear layer (INL), outer plexiform layer (OPL), and outer nuclear layer (ONL). We performed between-group comparisons of the postoperative thickness of each retinal layer as well as the postoperative change in the retinal thickness. The TRL was defined as the distance between the internal limiting membrane (ILM) and Bruch’s membrane. The IRL was defined as the distance between the ILM and upper border of the external limiting membrane (ELM). The ORL was defined as the distance between the upper border of the ELM and Bruch’s membrane.

The macular mapping protocol, which was the Early Treatment of Diabetic Retinopathy Study (ETDRS) grid of OCT, provided a scan length of 6 mm. Segmentation mode was used to divide the retinal layers; further, thickness map mode with ETDRS grid was used. The ETDRS grid divides the macula into nine regions defined by four quadrants (nasal, temporal, superior, inferior) and three rings (1-, 3-, and 6-mm diameters for the foveal center, inner macular ring, and outer macular ring, respectively). The average retinal thickness of the four inner macular ring quadrants were calculated (See Additional file 1).

### Statistical analysis

Statistical analyses were performed using R version 4.1.2 (R Foundation for Statistical Computing, Vienna, Austria). For each group, the mean and standard deviation of the baseline characteristic values including age, axial length, phacoemulsification time, CDE, CDVA, IOP, and spherical equivalent were calculated. Between-group comparisons of categorical and continuous variables were performed using the chi-square test and independent *t*-test, respectively. Furthermore, the mean and standard deviation of the measured thicknesses for each retinal layer were calculated. Paired *t* test was used for within-group comparisons of preoperative and postoperative retinal thickness. Multiple regression analysis was used for between-group comparisons of preoperative and postoperative retinal thickness values, as well as alterations in retinal thickness, with adjustment for age and axial length values. A *P*-value < 0.05 was considered statistically significant.

## Results

### Baseline characteristics

A total of 113 eyes were included; among them, 55 eyes of 35 patients underwent FLACS, while 58 eyes of 37 patients underwent CCS. The FLACS group comprised 17 (30.9%) men and 38 (69.1%) women, with a mean age of 68.71 ± 8.18 years (range: 39–79 years). The CCS group comprised 21 (36.2%) men and 37 (63.8%) women, with a mean age of 69.09 ± 6.25 years (range: 53–79 years). There were no significant between-group differences in age (*P* = 0.783), male to female ratio (*P* = 0.692), laterality (*P* = 1.000), axial length (*P* = 0.382), diagnosis of diabetes mellitus (*P* = 0.569), diagnosis of hypertension (*P* = 0.184), cataract grade (nuclear opacity grading based on the lens opacities classification system III) (*P* = 0.551), phacoemulsification time (*P* = 0.670), and CDE (*P* = 0.787). There were no significant between-group differences in preoperative and postoperative corrected distance visual acuity, intraocular pressure, and spherical equivalence (*P* > 0.05) (Table 1).

**Table 1:** Comparison of baseline characteristics of patients who underwent conventional or femtosecond-laser assisted cataract surgery

Feature	CCS (*n* = 58)		FLACS (*n* = 55)		*P* value	
Age (years)	69.09 ± 6.25		68.71 ± 8.18		0.783	
Gender (M/F)	21/37		17/38		0.692	
Laterality (R/L)	27/31		26/29		1.000	
Axial length (mm)	23.53 ± 0.80		23.69 ± 1.07		0.382	
DM (%)	22.4		16.4		0.569	
HTN (%)	44.8		30.9		0.184	
Nuclear opacity (LOCS III)	2.78 ± 0.90		2.87 ± 0.82		0.551	
Phacoemulsification time (sec)*	20.63 ± 15.09		21.91 ± 12.89		0.670	
CDE (sec)*	5.35 ± 4.26		5.57 ± 3.37		0.787	
	Preop	Postop	Preop	Postop	*P* value ^a^	*P* value ^b^
CDVA (decimal)	0.64 ± 0.22	0.98 ± 0.06	0.66 ± 0.23	0.98 ± 0.04	0.512	0.808
IOP (mmHg)	13.40 ± 2.56	12.34 ± 2.59	13.95 ± 2.97	12.51 ± 2.89	0.294	0.751
SE (diopter)	0.41 ± 1.92	-0.51 ± 0.85	0.40 ± 2.38	-0.35 ± 0.42	0.970	0.222

M = male; F = female; R = right, L = left; DM = diabetes mellitus; HTN = hypertension; LOCS III = lens opacities classification system III; CDE = cumulative dissipated energy; CDVA = corrected distance visual acuity; IOP = intraocular pressure; SE = spherical equivalence

* Phacoemulsification time and CDE values were measured in 38 and 50 eyes in CCS and FLACS group, respectively

^a^ Comparison of preoperative features between CCS and FLACS group

^b^ Comparison of postoperative features between CCS and FLACS group

Age, axial length, phacoemulsification time, CDE, CDVA, IOP, SE values are represented as mean ± standard deviation

### Between-group comparison of preoperative and postoperative retinal thickness values

**Fig. 1:**
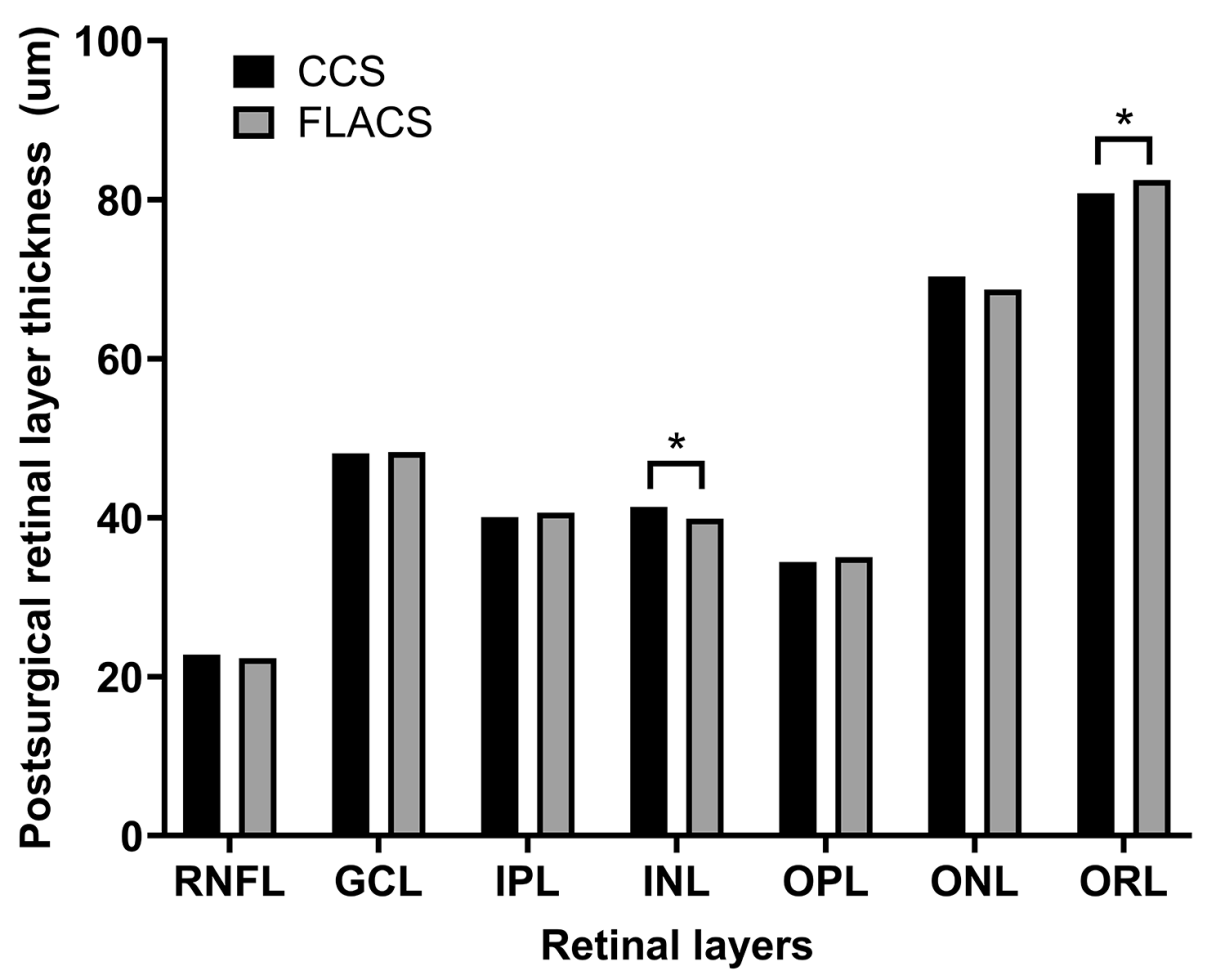
Between-group comparison of postsurgical retinal layer thickness

No significant between-group difference was noted in the preoperative thickness of each retinal layer. However, significant between-group differences were observed in the postoperative ORL (*P* = 0.004) and INL (*P* = 0.007) thickness, which showed opposite directions in the postoperative change. Specifically, compared with the CCS group, the FLACS group exhibited thicker ORL and thinner INL. There were no significant between-group differences in the postoperative thickness of the other retinal layers (Fig. 1; Table 2).

**Table 2:** Alterations in the thickness of retinal layers after surgery and comparison between the surgical groups

Retinal layers	Preop			Postop			Alteration					
	CCS (um)	FLACS (um)	*P* ^a^	CCS (um)	FLACS (um)	*P* ^a^	CCS (um)	*P*	FLACS (um)	*P*	Deviation (um) ^ab^	*P* ^a^
TRL	325.68 ± 17.14	327.15 ± 14.85	0.780	338.37 ± 19.74	336.96 ± 15.92	0.535	12.69	< 0.001	9.80	< 0.001	-2.78	0.027
IRL	243.41 ± 16.78	244.01 ± 14.30	0.953	257.45 ± 19.66	254.58 ± 14.85	0.274	14.04	< 0.001	10.57	< 0.001	-3.31	0.012
ORL	82.25 ± 3.18	83.17 ± 3.00	0.090	80.80 ± 3.13	82.46 ± 2.85	0.004	-1.44	< 0.001	-0.71	0.015	0.68	0.099
RNFL	21.03 ± 2.29	20.92 ± 2.47	0.568	22.78 ± 2.84	22.34 ± 3.00	0.239	1.75	< 0.001	1.41	< 0.001	-0.37	0.212
GCL	44.63 ± 7.10	45.46 ± 5.78	0.572	48.09 ± 7.50	48.26 ± 6.03	0.988	3.47	< 0.001	2.80	< 0.001	-0.62	0.066
IPL	38.13 ± 3.99	38.96 ± 3.61	0.278	40.07 ± 4.40	40.63 ± 3.95	0.531	1.94	< 0.001	1.67	< 0.001	-0.24	0.324
INL	39.72 ± 2.53	38.83 ± 2.64	0.056	41.35 ± 2.79	39.89 ± 2.79	0.007	1.63	< 0.001	1.05	< 0.001	-0.53	0.103
OPL	35.59 ± 6.16	36.69 ± 6.76	0.472	34.43 ± 6.37	35.04 ± 6.59	0.762	-1.16	0.013	-1.65	0.014	-0.50	0.528
ONL	64.10 ± 9.10	63.21 ± 6.78	0.558	70.35 ± 9.82	68.70 ± 7.35	0.355	6.25	< 0.001	5.49	< 0.001	-0.64	0.483

TRL = total retinal layer; IRL = inner retinal layer; ORL = outer retinal layer; RNFL = retinal nerve fiber layer; GCL = ganglion cell layer; IPL = inner plexiform layer; INL = inner nuclear layer; OPL = outer plexiform layer; ONL = outer nuclear layer

^a^ Age and axial length adjusted

^b^ The deviation of postoperatively altered retinal thickness values in the FLACS group compared to the CCS group

Retinal thickness values are represented as mean ± standard deviation

### Postoperative change in each retinal layer in the CCS and FLACS groups

**Fig. 2:**
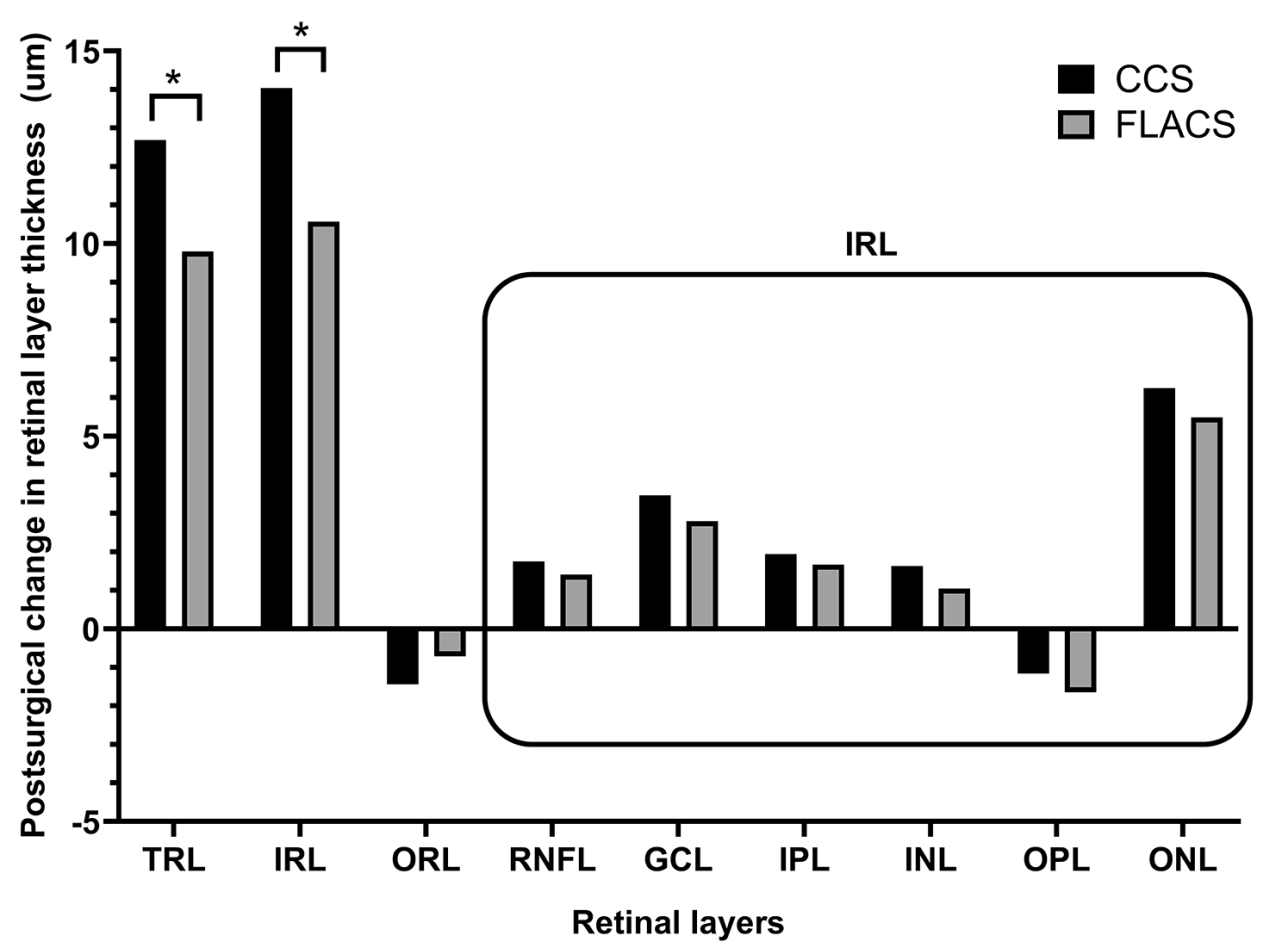
Between-group comparison of postsurgical changes in retinal layer thickness

In both groups, all retinal layer thickness values showed significant changes at 1 postoperative month; further, the change direction for each retinal layer thickness was identical between the groups. Specifically, a significant increase was noted in the thickness of the TRL, IRL, RNFL, GCL, IPL, INL, and ONL (*P* < 0.001), but a significant decrease was observed in the thickness of the ORL and OPL (*P* < 0.001, *P* = 0.013) (Fig. 2; Table 2).

### Between-group comparison of changes in each retinal layer thickness

We performed between-group comparisons of postoperative changes in retinal layer thickness values. There were significant between-group differences in the postoperative change in the TRL and IRL thickness (*P* = 0.027 and *P* = 0.012, respectively), but not in the RNFL, GCL, IPL, INL, OPL, ONL, and ORL thickness (*P* > 0.05). Adjustment for age and axial length in the regression model yielded the same results (*P* > 0.05). The postoperative increase in TRL and IRL thickness was significantly smaller in the FLACS group than in the CCS group (Table 2; Fig. 2). After adjustment for the postoperative change in IRL thickness, there was no significant between-group difference in the postoperative change in TRL thickness (*P* = 0.527). This indicated that the IRL change substantially influenced the observed between-group differences in TRL changes (*P* < 0.001).

## Discussion

We observed substantial alterations in the thickness of each retinal layer following both CCS and FLACS. Both groups showed a postoperative increase and decrease in the thickness of the IRL and ORL, respectively. Accordingly, the TRL thickness increased due to the more notable increase in the thickness of the IRL. Specifically, the thickness of each retinal layer comprising the IRL, except the OPL, showed a postoperative increase. Notably, the postoperative alteration patterns of the retinal layer thickness were identical between the CCS and FLACS group. Although we observed significant between-group differences in the postoperative thickness of the ORL and INL, there were no between-group differences in the postoperative changes in the thickness of individual retinal layers; specifically, each of the six layers comprising the IRL. However, the cumulative change across the six layers, specifically the IRL, showed a significant between-group difference, indicating that the combination of nonsignificant changes in each layer yielded a meaningful distinction (Fig. 2).

The between-group difference in the postoperative change in TRL thickness could be mostly attributed to the between-group difference in the alteration of the IRL thickness. After adjustment of the postoperative change in IRL thickness, there was no between-group difference in the postoperative change in TRL thickness (*P* = 0.527), even after adjustment for age and axial length (*P* = 0.580). Additionally, the IRL change was identified as a factor substantially contributing to the observed between-group differences in the postoperative change in TRL thickness (*P* < 0.001). Taken together, the postoperative change in IRL thickness can be considered as the primary factor with a between-group difference, which subsequently contributed to the between-group difference in the TRL thickness.

Several studies have reported no notable difference in retinal thickness after one postoperative month between individuals who underwent CCS and FLACS [17, 19–25]. However, Wang et al. reported that the foveal volume and average retinal thickness was significantly increased following conventional phacoemulsification cataract surgery, but not after FLACS [17]. Moreover, another study has reported lower postoperative macular thickness in the FLACS group than in the CCS group. Specifically, although the FLACS group showed lower preoperative macular thickness, the CCS group showed a larger mean increase in macular thickness, which further accentuates the between-group difference in postoperative macular thickness [18]. These previous reports are consistent with our findings, indicating that FLACS has a lesser impact on the retina than CCS.

Cataract surgery is an established risk factor for subclinical macular edema, [10] and the CME predominantly affects the inner retinal layers, specifically the INL [26]. Muller cells, which are primary cells in the INL, are susceptible to fluid accumulation, resulting in the development of cystic spaces indicative of CME. Given the presence of two diffusion barriers, the IPL and OPL, the distribution of intraretinal fluid is constrained. Consequently, when serum leaks from intraretinal vessels, it primarily leads to cyst development within the INL. This may explain the more noticeable postoperative alterations in the IRL than in the ORL observed in our study (Fig. 2).

Although further evidence is warranted, the surgical impact may induce edematous changes in the INL and neighboring retinal layers, leading to a relative reduction in the ORL thickness due to pressure effects. The relative decrease in ORL thickness could involve the barrier function and dynamic transport mechanism within the retinal pigment epithelium [27]. 

Our findings indicate that FLACS had a less significant effect on retinal thickness than CCS. Both groups showed a postoperative decrease and increase in ORL and INL thickness, respectively. However, compared with CCS group, the FLACS group showed higher and lower postoperative ORL and INL thickness, respectively (Fig. 1). This indicates that the postoperative alterations were comparatively milder after FLACS than after CCS. Moreover, the degree of increase in the TRL and IRL thickness was less pronounced in the FLACS group than in the CCS group (Fig. 2). As the INL and surrounding retinal layers tend to become swollen after surgery, FLACS can be considered to involve less postoperative alterations in the retinal layers. Although the difference might be regarded as subclinical change given the resolution limits of OCT, [28] the consistent postoperative retinal change and notable difference between the two groups implies that there is an underlying molecular-level distinction causing it. This subclinical difference should not be overlooked because this can lead to substantial consequences, particularly in scenarios more susceptible to causing notable postoperative CME.

The mechanism underlying postsurgical macular edema remains unclear. However, the release of inflammatory mediators has been proposed to be involved [9, 12, 14]. There is strong evidence indicating that FLACS involves less tissue damage and milder inflammatory responses than CCS [29–35]. Specifically, Abell et al. demonstrated that anterior segment inflammation was less after FLACS than after manual cataract surgery [30]. Moreover, FLACS has been shown to involve lower ECC loss than conventional phacoemulsification [31, 33–35]. The reduced phacoemulsification process in FLACS has been suggested to contribute to the reduced tissue damage and decreased inflammatory response in FLACS [15, 33, 36]. However, we observed no significant between-group differences in the phacoemulsification time and CDE. Nevertheless, there have been extensive reports of discrepancies in inflammatory indicators, even without reported differences in phacoemulsification parameters [17, 20, 21, 32, 34, 35]. 

This study has few limitations. First, this was a small-scale, single-center study with a short follow-up duration. Second, retinal thickness can be affected by circadian rhythm to a small extent, [37] so controlling for this would have allowed for a more accurate comparison of results. However, since all patients underwent OCT in the early afternoon, the time difference between patients should not be more than 3 h at most. Moreover, since we did not intentionally control for this variable, we believe that randomization was achieved. Third, given the missing data for some cases, our findings regarding the phacoemulsification parameters should be interpreted with caution. Nevertheless, this study represents the first comparative analysis of altered retinal thickness values between the FLACS and CCS groups. Specifically, this study incorporates data on the changes in thickness of each retinal layer, offering insights into molecular-level alterations in the retina, which is a novel aspect of this study.

## Conclusions

In conclusion, the overall postsurgical effect was less pronounced in the FLACS group than in the CCS group. In the FLACS group, the postoperative thickness of the ORL and INL was higher and lower, respectively. The postoperative increase in the IRL thickness was less pronounced in the FLACS group than in the CCS group. These findings indicate that FLACS involves a relatively minor inflammatory response due to reduced tissue damage. Further studies are warranted to explore the impact of FLACS on the retina and to elucidate the underlying mechanisms.

### Electronic supplementary material

Below is the link to the electronic supplementary material.

**Additional file 1.** Four inner macular ring quadrants

## Data Availability

The datasets generated during and/or analyzed during the current study are available from the corresponding author upon reasonable request.

## Abbreviations

CCS: Conventional cataract surgery
CDE: Cumulative dissipated energy
CDVA: Corrected distance visual acuity
CME: Cystoid macular edema
ECC: Endothelial cell count
ELM: External limiting membrane
ETDRS: Early Treatment of Diabetic Retinopathy Study
FLACS: Femtosecond laser-assisted cataract surgery
GCL: Ganglion cell layer
ILM: Internal limiting membrane
INL: Inner nuclear layer
IOP: Intraocular pressure
IPL: Inner plexiform layer
IRL: Inner retinal layer
OCT: Optical coherence tomography
ONL: Outer nuclear layer
OPL: Outer plexiform layer
ORL: Outer retinal layer
PG: Prostaglandin
RNFL: Retinal nerve fiber layer
TRL: Total retinal layer

## References

[1] Friedman NJ, Palanker DV, Schuele G, Andersen D, Marcellino G, Seibel BS (2011). Femtosecond laser capsulotomy. J Cataract Refract Surg.

[2] Nagy ZZ, Kranitz K, Takacs AI, Mihaltz K, Kovacs I, Knorz MC (2011). Comparison of intraocular lens decentration parameters after femtosecond and manual capsulotomies. J Refract Surg.

[3] Mihaltz K, Knorz MC, Alio JL, Takacs AI, Kranitz K, Kovacs I (2011). Internal aberrations and optical quality after femtosecond laser anterior capsulotomy in cataract surgery. J Refract Surg.

[4] Kranitz K, Mihaltz K, Sandor GL, Takacs A, Knorz MC, Nagy ZZ (2012). Intraocular lens tilt and decentration measured by Scheimpflug camera following manual or femtosecond laser-created continuous circular capsulotomy. J Refract Surg.

[5] Kranitz K, Takacs A, Mihaltz K, Kovacs I, Knorz MC, Nagy ZZ (2011). Femtosecond laser capsulotomy and manual continuous curvilinear capsulorrhexis parameters and their effects on intraocular lens centration. J Refract Surg.

[6] Roberts HW, Wagh VK, Sullivan DL, Archer TJ, O’Brart DPS (2018). Refractive outcomes after limbal relaxing incisions or femtosecond laser arcuate keratotomy to manage corneal astigmatism at the time of cataract surgery. J Cataract Refract Surg.

[7] Ruckl T, Dexl AK, Bachernegg A, Reischl V, Riha W, Ruckhofer J (2013). Femtosecond laser-assisted intrastromal arcuate keratotomy to reduce corneal astigmatism. J Cataract Refract Surg.

[8] Schultz T, Joachim SC, Kuehn M, Dick HB (2013). Changes in prostaglandin levels in patients undergoing femtosecond laser-assisted cataract surgery. J Refract Surg.

[9] Makri OE, Georgalas I, Georgakopoulos CD (2013). Drug-induced macular edema. Drugs.

[10] Sorr EM, Everett WG, Hurite FG (1979). Incidence of fluorescein angiographic subclinical macular edema following phacoemulsification of senile cataracts. Ophthalmology.

[11] Chambless WS (1979). Phacoemulsification and the retina: cystoid macular edema. Ophthalmology.

[12] Zur D, Loewenstein A (2017). Postsurgical Cystoid Macular Edema. Dev Ophthalmol.

[13] Flach AJ (1998). The incidence, pathogenesis and treatment of cystoid macular edema following cataract surgery. Trans Am Ophthalmol Soc.

[14] Miyake K, Ibaraki N (2002). Prostaglandins and cystoid macular edema. Surv Ophthalmol.

[15] Nagy Z, Takacs A, Filkorn T, Sarayba M (2009). Initial clinical evaluation of an intraocular femtosecond laser in cataract surgery. J Refract Surg.

[16] Kanclerz P, Alio JL (2021). The benefits and drawbacks of femtosecond laser-assisted cataract surgery. Eur J Ophthalmol.

[17] Wang Y, Zhang J, Qin M, Miao J, Chen W, Huang Y (2020). Comparison of optical quality and distinct macular thickness in femtosecond laser-assisted versus phacoemulsification cataract surgery. BMC Ophthalmol.

[18] Renones J, Anton A, Gonzalez-Martin JM, Carreras H, Loro-Ferrer JF (2023). Effect of conventional cataract surgery and Femtosecond Laser-assisted cataract surgery on Bruch’s membrane opening-minimum Rim Width, retinal nerve Fiber layer, and Macular Thickness. J Ophthalmol.

[19] Slezak F, Thumann G, Kropp M, Cvejic Z, De Clerck EEB, Bravetti GE et al. Comparison of Conventional and Femtosecond Laser-assisted cataract surgery regarding Macula Behavior and Thickness. Med (Kaunas). 2023;59(4). 10.3390/medicina59040639 PMC10145739 37109597

[20] Mursch-Edlmayr AS, Bolz M, Luft N, Ring M, Kreutzer T, Ortner C (2017). Intraindividual comparison between femtosecond laser-assisted and conventional cataract surgery. J Cataract Refract Surg.

[21] Ecsedy M, Mihaltz K, Kovacs I, Takacs A, Filkorn T, Nagy ZZ (2011). Effect of femtosecond laser cataract surgery on the macula. J Refract Surg.

[22] Asena BS, Karahan E, Kaskaloglu M (2017). Retinal and choroidal thickness after femtosecond laser-assisted and standard phacoemulsification. Clin Ophthalmol.

[23] Conrad-Hengerer I, Hengerer FH, Al Juburi M, Schultz T, Dick HB (2014). Femtosecond laser-induced macular changes and anterior segment inflammation in cataract surgery. J Refract Surg.

[24] Schweitzer C, Brezin A, Cochener B, Monnet D, Germain C, Roseng S (2020). Femtosecond laser-assisted versus phacoemulsification cataract surgery (FEMCAT): a multicentre participant-masked randomised superiority and cost-effectiveness trial. Lancet.

[25] Day AC, Burr JM, Bennett K, Bunce C, Dore CJ, Rubin GS (2020). Femtosecond Laser-assisted cataract surgery Versus Phacoemulsification cataract surgery (FACT): a Randomized Noninferiority Trial. Ophthalmology.

[26] Bringmann A, Reichenbach A, Wiedemann P (2004). Pathomechanisms of cystoid macular edema. Ophthalmic Res.

[27] Yang S, Zhou J, Li D (2021). Functions and diseases of the retinal pigment epithelium. Front Pharmacol.

[28] Spaide RF, Otto T, Caujolle S, Kubler J, Aumann S, Fischer J (2022). Lateral resolution of a Commercial Optical Coherence Tomography Instrument. Transl Vis Sci Technol.

[29] Favuzza E, Becatti M, Gori AM, Mencucci R (2019). Cytokines, chemokines, and flare in the anterior chamber after femtosecond laser-assisted cataract surgery. J Cataract Refract Surg.

[30] Abell RG, Allen PL, Vote BJ (2013). Anterior chamber flare after femtosecond laser-assisted cataract surgery. J Cataract Refract Surg.

[31] Khan MS, Habib A, Ishaq M, Yaqub MA (2017). Effect of Femtosecond Laser-assisted cataract surgery (FLACS) on endothelial cell Count. J Coll Physicians Surg Pak.

[32] Takacs AI, Kovacs I, Mihaltz K, Filkorn T, Knorz MC, Nagy ZZ (2012). Central corneal volume and endothelial cell count following femtosecond laser-assisted refractive cataract surgery compared to conventional phacoemulsification. J Refract Surg.

[33] Krarup T, Ejstrup R, Mortensen A, la Cour M, Holm LM (2019). Comparison of refractive predictability and endothelial cell loss in femtosecond laser-assisted cataract surgery and conventional phaco surgery: prospective randomised trial with 6 months of follow-up. BMJ Open Ophthalmol.

[34] Chee SP, Yang Y, Wong MHY (2021). Randomized controlled trial comparing Femtosecond Laser-assisted with conventional phacoemulsification on dense cataracts. Am J Ophthalmol.

[35] Liu YC, Setiawan M, Chin JY, Wu B, Ong HS, Lamoureux E (2021). Randomized controlled trial comparing 1-Year outcomes of low-energy Femtosecond Laser-assisted cataract surgery versus conventional phacoemulsification. Front Med (Lausanne).

[36] Cavallini GM, Fornasari E, De Maria M, Lazzerini A, Campi L, Verdina T (2019). Bimanual femtosecond laser-assisted cataract surgery compared to standard bimanual phacoemulsification: a case-control study. Eur J Ophthalmol.

[37] Ashraf H, Nowroozzadeh MH (2014). Diurnal variation of retinal thickness in healthy subjects. Optom Vis Sci.

