# Supplementary figures and images for: Comparative analysis of changes in retinal layer thickness following femtosecond laser-assisted cataract surgery and conventional cataract surgery
Source: BMC Ophthalmol. 2024 Jul 9;24:276. doi: 10.1186/s12886-024-03543-1 (PMC11232152; doi:10.1186/s12886-024-03543-1)

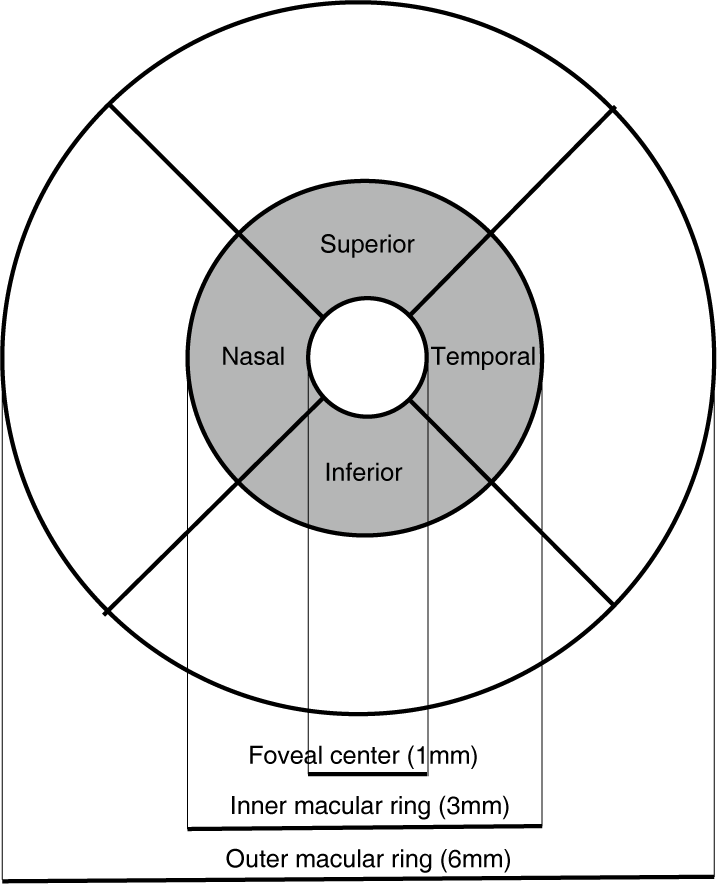

Supplement: Supplementary file 1 — Additional file 1. Four inner macular ring quadrants [file 12886_2024_3543_MOESM1_ESM.tif]
